# Coordinated Increased Expression of Cyclooxygenase2 and Nuclear Factor *κ*B Is a Steady Feature of Urinary Bladder Carcinogenesis

**DOI:** 10.1155/2010/871356

**Published:** 2010-08-23

**Authors:** Stylianos Kontos, Georgia Sotiropoulou-Bonikou, Athina Kominea, Maria Melachrinou, Eleni Balampani, Dionysis Bonikos

**Affiliations:** ^1^Department of Pathology, Medical School, University of Patras, 26504 Rion, Greece; ^2^Department of Urology, General Hospital of Nikaia, 18543 Peiraeus, Greece; ^3^Department of Anatomy and Histology-Embryology, Medical School, University of Patras, 26504 Rion, Greece; ^4^Department of Pathology, Aegion General Hospital, 25100 Aegion, Greece; ^5^Department of Microbiology, General Hospital of Nikaia, 18543 Peiraeus, Greece

## Abstract

*Objectives*. The inescapable relationship between chronic inflammation and carcinogenesis has long been established. Our objective was to investigate COX-2 and NF-*κ*B immunohistochemical expression in a large series of normal epithelium and bladder carcinomas. *Methods*. Immunohistochemical methodology was performed on formalin-fixed, paraffin-embedded sections from urinary bladder carcinomas of 140 patients (94 males and 46 females with bladder carcinomas). *Results*. COX-2 expression is increased in the cytoplasm of bladder cells, during loss of cell differentiation (*r*_s_ = 0.61, *P*-value < .001) and in muscle invasive carcinomas (*P*-value < .001). A strong positive association between tumor grade and nuclear expression of NF*κ*B has been established. A positive correlation between COX-2 and nuclear NF*κ*B immunoreactivity was observed. *Conclusions*. The possible coordinated upregulation of NF*κ*B and COX-2, during bladder carcinogenesis, indicates that agents inhibitors of these two molecules may represent a possible new treatment strategy, by virtue of their role in bladder carcinogenesis.

## 1. Introduction

Urothelial carcinoma (UC) of the urinary bladder is the third most common cancer in men and the 15th most common cancer in women. UC-related deaths are mainly caused by the invasive type of the disease [1]. However, the more frequent form of this carcinoma is either non-invasive or superficially invasive disease, which is usually curable, but demonstrates a challenge to the clinician because of its recurrent nature. Thus, more effective therapies are needed to prevent recurrence of superficial UC and to inhibit progression of noninvasive tumors to invasive carcinomas [2]. 

The inescapable relationship between chronic inflammation and carcinogenesis has long been established and rests on DNA damage. Chronic inflammation is now recognized as crucial in the pathogenesis of a variety of diseases, such as arthritis, diabetes, atherosclerosis, Alzheimer's disease, and cancer development, including bladder cancer [3]. To better understand the importance of chronic inflammation in bladder cancer pathogenesis and progression, we investigated COX-2 and NF-*κ*B p65 immunohistochemical expression in a large series of normal epithelium and bladder carcinomas, and we correlated those findings with cancer cell differentiation, tumor grade and stage, and clinical-pathologic features of bladder cancer patients.

Cyclooxygenase-2 (COX-2) is an enzyme missing from most healthy tissues, while its presence is well detected in neoplastic and inflammatory foci [4]. As a catalyst of prostaglandin synthesis, it promotes the aggregation of inflammatory mediators. COX-2 is a phase I enzyme, which converts inert xenobiotic ingredients of tobacco smoke into carcinogenic substances [5]. Finally, tumor angiogenesis is promoted via prostaglandin action. Regulation of COX-2 expression is another field of interest. Two signalling pathways are intensely studied: the mitogen-activated protein kinase cascade is activated by numerous extra- or intracellular events, culminating in COX-2 transcription [6]. The second pathway begins following either interleukin-1 or tumor-necrosis-factor-mediated cell stimulation, and terminates with recruitment of Nuclear Factor-*κ*B (NF-*κ*B) [4].

NF*κ*B is a transcription factor that regulates a multitude of inflammatory genes, including cytokines, chemokines, adhesion molecules, and acute phase proteins [7]. NF*κ*B is a complex of two variable subunits of the Rel/NF*κ*B superfamily RelA(p65), RelB, c-Rel, p100/p52(NF*κ*B 2), and p105/p50 NF*κ*B. In most cell types, NF*κ*B dimmers remain in the cytoplasm transcriptionally silent by virtue of being bound to an inhibitor protein called Inhibitor-Kappa-B (I-*κ*B) [8]. Activation of I*κ*B kinase in response to extracellular stimuli, such as oxidative stress, ischemia, bacteria, and viruses, leads to I*κ*B phosphorylation and degradation [9]. The liberated NF*κ*B translocates to the nucleus, where it actively participates in transcription of a plethora of genes, involved in proliferation, cell survival, cell adhesion and encoding cytokines, chemokines, cyclin D, matrix metalloproteinase, factors of angiogenesis and antiapoptotic proteins [10]. Molecular studies in multiple myeloma [11], lymphoma [12], breast [13], pancreatic [14], prostate cancer [15], head and neck squamous cell carcinoma [16], and colon [17] and bladder cancer [18] have demonstrated that NF*κ*B may have a critical role in cancer development and chemoresistance [19].

Carcinogenesis is a complicated multistep process that gradually deprives normal cells of their natural phenotype, resulting in tissue disturbance, from which tumor finally emerges. The understanding of the molecular mechanisms involved in the pathogenesis and progression of bladder cancer may lead to new and more effective strategies for risk assessment, prevention, early detection, and targeted treatment [20].

## 2. Materials and Methods

### 2.1. Clinical Specimens

140 consecutive patients diagnosed with urothelial carcinoma (UC) of the urinary bladder by either biopsies, transurethral resection of bladder tumor (TURBT), or cystectomies, at the Department of Urology, School of Medicine, University of Patras, Greece, between 2000 and 2002, were selected for this study. Demographic information indicated the presence of 94 males and 46 females with a median age of 70 years (range, 23–90 years). None of the patients had received any preoperative intravesical treatment or systemic chemotherapy. The research was approved by the University of Patras Institutional Review Board and by the Local Research Ethics Committee at University Hospital of Patras, according to the principles laid down by the Declaration of Helsinki.

Tumors were graded according to the World Health Organization Classification. (1973 WHO grading) [21], even outdated but urologists are more familiar with that grading system, and T classification [22]. Staging was based on combination of clinical (cytoscopy, computed tomography, and ultrasound) and histological data to determine the absence (Ta) or presence (T1) of invasion of the lamina propria or invasion into or beyond the detrusor muscle (T2–T4) as displayed in Table 1.

### 2.2. Immunohistochemistry

Tissues were fixed in 10%(v/v) buffered formalin and paraffin embedded and stored in the Department of Pathology, School of Medicine, University of Patras. Serial 5 *μ*m sections were obtained for immunohistochemical staining and were mounted on positively charged slides. The primary antibodies used were a mouse monoclonal IgG1anti-NF*κ*B subunit p65 (anti-NF*κ*B p65 (F-6) Santa Cruz Biotechnology, USA), and a rabbit polyclonal anti-COX-2 antibody (anti-COX-2, (Sc-7951) Santa Cruz Biotechnology, USA).

Sections were restored at 37°C for 24 hours and dewaxed in xylene and ethanol, using serial concentrations, 100-96-80 for 5 minutes each. Endogenous peroxidase activity was blocked with 0.3% hydrogen peroxide for 15 minutes. Antigen retrieval was then performed in citrate buffer (0.001 M, ph 6) by microwaving as follows: 2.5 mins at 500 w, 2.5 mins at 700 w, and 10 mins at 300 W. After cooling to room temperature for 20 mins, sections were incubated with blocking serum in TBS (Tris buffered saline) for another 20 mins. Afterwards sections were incubated with CAS block (Zymed Laboratories Inc, San Francisco, California, USA) for 15 mins, in order to reduce nonspecific staining, and then with the primary antibody for 30 mins (diluted 1 : 100 for p65 and 1 : 100 for COX-2), and finally with the Super Picture Kit (Zymed) for 50 mins. For both antigens, DAB and hematoxylin for the nuclear staining were used. Negative controls were prepared, substituting Tris-buffered saline for primary antibody. Known immunostaining positive specimens were used as a positive controls for NF*κ*B and COX-2.

### 2.3. Evaluation of Immunostaining

Regarding normal transitional cell epithelium, every effort was made to obtain data from patients with free medical history, who underwent biopsies from suspicious lesions (that turned to have no histopathological abnormalities) of bladder epithelium after cystoscopy, for investigation of an episode of hematuria. Immunostained sections were examined under high-power (x400) microscopy. COX-2 displays cytoplasmic immunoreactivity and p65 subunit of NF*κ*B is either cytoplasmic or nuclear or both depending on the state of activation. Therefore, the two cellular compartments were examined separately. Immunostained sections were evaluated on five (5) different representative microscopic fields from each section at 400x magnification. At least 1000 cells were counted in tissue sections obtained from carcinomas. In tissue sections obtained from normal bladder epithelium, 200–300 cells were counted. For each field, a percentage (%) of positive cells was calculated and the average of those was taken. The intensity of the immunohistochemical staining was scored on a four-point scale as follows: − = negative (<10% of cells with nuclear staining), + = weak (weak nuclear staining intensity or 10%–50% of cells with nuclear staining), ++ = moderate (moderate nuclear staining intensity and >50% of cells with nuclear staining), and +++ = strong expression (strong nuclear staining intensity and >50% of cells with nuclear staining) [23]. Two independent pathologists evaluated and scored all sections using the scale without prior knowledge of the clinicopathologic characteristics of each case. When interobserver disagreement was observed, specimens were reassessed by simultaneous examination by the two pathologists in a double-headed light microscope. There was no interobserver disagreement regarding assessments of tumor differentiation.

### 2.4. Statistical Analysis

Categorical variables are presented as absolute and relative frequencies. Chi-square test (Pearson or Fisher's exact) were used to evaluate the relationship between the histological grade stage and NF*κ*B expression, grade stage and COX-2 expression, and grade stage and gender (to assess the association between histological grade and gender and also age). All reported *P* values were based on two-sided hypothesis. *P* value less than .05 were considered statistically significant. Spearman rank correlation coefficient (*r*) was computed in order to estimate the direction and strength of correlations between histological grade and NF*κ*B-COX-2 expressions. Also (*r*) was used for assessing the relationship between NF*κ*B and COX-2 expressions. The above-mentioned statistical methods were also applied separately according to gender or to age groups (<70 years and ≥70 years of age).

The Cochran-Armitage test for trend was applied in NF*κ*B and COX-2 in order to examine possible trends of the two expressions across the ordered levels of tumor grade stage. The above mentioned statistical methods were also applied separately according to gender or age groups (<70 years and ≥70 years of age). SPSS software was used for all analyses (SPSS Inc., 2003, Chicago,5 IL USA).

## 3. Results and Discussion

**Figure 1:**
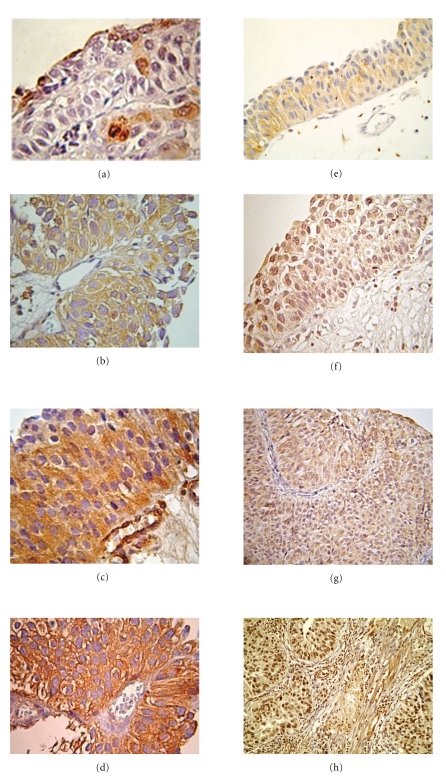
(a) and (e)  Normal bladder epithelium. (a) Epithelial bladder cells exhibit no immunostaining for COX-2 (x200). (e) Strong positive cytoplasmic and negative nuclear expression for NF*κ*B (x200). (b) and (f) Well-differentiated UC (Grade I) (b) Epithelial bladder cells exhibit weak positive cytoplasmic immunostaining for COX-2 (x200). (f) Moderate nuclear and strong cytoplasmic immunostaining for NF*κ*B (x200). (c) and (g) Moderately differentiated UC (Grade II). (c) Epithelial bladder cells exhibit moderate cytoplasmic immunostaining for COX-2 (x200). (g) Strong nuclear and moderate cytoplasmic immunostaining for NF*κ*B (x200). (d) and (h) Poorly differentiated UC (Grade III). (d) Epithelial bladder cells exhibit strong cytoplasmic immunostaining for COX-2 (x200). (h) Strong nuclear immunostaining for NF*κ*B (x200).

**Figure 2:**
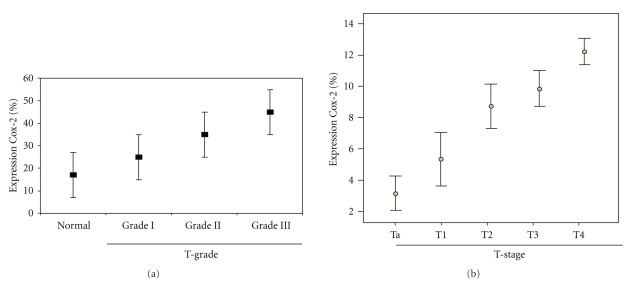
COX-2 in 140 patients participating in the study stratified by (a) tumor grade and (b) tumor stage. (error bars = standard error of the value).

COX-2 was consistently detected in the cytoplasm of epithelial cells (Figures 1(a)–1(d)). Cytoplasmic staining of COX-2 manifested a noteworthy variation among histological categories as depicted in Table 2 and Figure 2(a). Only 9.3% (5/54) of Grade III carcinomas lacked COX-2 positivity whereas 43.4% (23/54) and 18.9% (11/54) of specimens manifested strong immunostaining. A positive statistical significant correlation was established between tumor grade and expression of COX-2 (*r*_s_ = 0.24, *P*-value < .001). Only 17.2% (5/29) of normal bladder epithelium presented with strong nuclear COX-2 immunoreactivity. COX-2 staining increased with increasing histological stage in the entire cohort. Specifically COX-2 expression was positively correlated with T-tumor category and in muscle invasive carcinomas (*r*_s_ = 0.61, *P*-value < .001) (Figure 2(b)). Intergroup comparisons, regarding correlation of clinicopathological parameters with expression levels of COX-2 were performed. There was no statistically significant correlation. 

**Figure 3:**
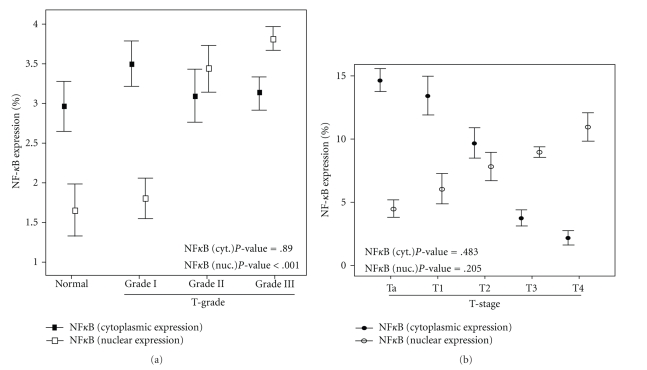
NF*κ*B nuclear (white box) and cytoplasmic (black box) expression, stratified by (a) tumor grade and (b) tumor stage. Nuclear expression of NF*κ*B was more frequent, during tumor dedifferentiation. (error bars = standard error of the value).

NF*κ*B epithelial staining was both cytoplasmic (Figures 1(e)–1(f)) and nuclear (Figures 1(g)–1(h)). The immunohistochemical expression of NF*κ*B in the cytoplasm varied according to histologic type and values of immunohistochemical scores for each grade of differentiation, as depicted in Table 3. Statistical analysis revealed no correlation between cytoplasmic expression and tumor grade (Figure 3(a)). Specifically, as normal cells progressively gained atypical characteristics, the cytoplasmic expression of p65 has been downregulated, without statistical confirmation (Spearman correlation coefficient *r* = −0.012, *P*  value = .890). 

As far as nuclear staining of NF*κ*B is concerned, a positive correlation between expression and progression of carcinogenesis was established. Normal epithelium specimens lacked NF*κ*B nuclear staining (Figure 3(a)). The correlation of molecular expression and tumor grades was investigated by the Spearman rank correlation coefficient test, which revealed a strong positive association between histological grade and nuclear expression of NF*κ*B (*P*  value < .001). No correlation was found between nuclear (*P*  value = .205) and cytoplasmic staining (*P*  value = .483) of NF*κ*B and tumor stage (Figure 3(b)). When we compared values concerning male and female patients, no statistical significance was established either between them (*P*  value = .675), or between the two age groups (*P*  value = .75). 

We further evaluated the correlation between the expression of COX-2 and NF*κ*B, separately for cytoplasmic and nuclear localization**.** When taking into account the total number of patients participating in this study, no statistically significant association (*r*_s_ = 0.11, value = .208) was obtained when correlating nuclear staining of NF*κ*B and cytoplasmic of COX-2. Similarly, cytoplasmic staining of NF*κ*B had no correlation with COX-2 (*r*_s_ = −0.01, *P*  value = .90). With regard to the comparison of cytoplasmic expression of NF*κ*B and COX-2, no correlation was established (Table 4).

The present retrospective immunohistochemical study demonstrated the expression of NF*κ*B and COX-2 in normal bladder urothelium and urothelial carcinomas and examined the variation of its expression. We extended our investigation along the tumor grades, using the World Health Organization (WHO) 1973 grading system, and showed how the expression of these factors varied along with T-stage, according to demographic data, gender, and age.

COX-2 expression was significantly higher in poorly differentiated cells, and this progressive upregulation was parallel with the loss of cancer cell differentiation. Accumulated epidemiological data suggest that COX-2 overexpression and enhanced prostaglandin synthesis are characteristics of quite a few premalignant and malignant conditions throughout the body [24]. Expression of COX-2 is common in advanced bladder carcinomas with poor prognostic characteristics, supporting efforts to initiate clinical trials on the efficacy of COX-2 inhibitors in the adjuvant treatment of high-risk urinary bladder cancer [25]. In detail, COX-2 increases during initiation and promotion phases from normal epithelium to carcinomas, exhibits a second rise synchronously with loss of cell differentiation, and reaches its highest expression as cells acquire invasive and metastatic properties. Persistent expression of COX-2 was noted in a similar study, since Cyclooxygenase-2 is not expressed in normal bladder urothelium, and COX-2 overexpression is associated with pathological and molecular features of biologically aggressive disease, suggesting a role for cyclooxygenase-2 in bladder cancer development and invasion [26]. On the whole, persistent expression of COX-2 in aberrant cells of premalignant forms leads to prolonged synthesis of carcinogenic factors, which, via the mechanisms described in the introductory section, slow down the healing effect of the self-repairing apparatus [27].

Next we extended our study, to the expression of NF*κ*B, the “master” regulator of chronic inflammation and malignant transformation [28]. Our results indicate an induction of this key molecule along the carcinogenesis path and the level of differentiation (Figures 1(e)–1(h)). We observed that normal urothelium displayed mainly cytoplasmic p65/RelA staining whereas in cancer cells p65 was seen in the nucleus. There was a progressive induction in nuclear p65 subunit of activated NF*κ*B expression that paralleled the loss of cancer cell differentiation, in higher tumor grade and advanced T-category whereas cytoplasmic staining decreased. Our data are consonant with previous studies, which also detected an induced expression of NF*κ*B in UC [18, 29] and in gastric, colon, and prostatic adenocarcinoma cells [30], when compared to their normal counterparts. The detailed mechanism underlying NF*κ*B activation in bladder cancer remains unclear. NF*κ*B activation is a multistep process in which several molecules interact to initiate a highly coordinated response [31]. The NF*κ*B-inducing kinase in combination with the increased activity of the IKK kinase complex could be the possible signaling pathway that results in the constitutive activation of the NF*κ*B protein in bladder carcinomas [32]. Concerning the association with gender and age, p65 content of male and female increased following the progress of carcinogenesis, with statistical strong correlation, but no difference was revealed with the comparison of genders or age (<70–>70).

A further aim of our study was to correlate the presence of the COX-2 enzyme with the expression of the regulatory factor NF-*κ*B. No association between COX-2 and cytoplasmic or nuclear p65 immunoreactivity was observed. Yet, several lines of evidence strengthen our suggestion that there is a positive correlation of COX-2 level and the nuclear fraction of p65, since these molecules are concurrently expressed, during carcinogenesis. Obviously, we recognize the limitations of such a correlation, since the amounts of both proteins are also associated with the same parameter, which is histological type. Esophageal carcinomas, originating from either squamous cell or Barrett's epithelium, exhibit coexpression of COX-2 and NF-*κ*B [33]. Moreover, Nadjar et al. [34] have demonstrated NF-*κ*B-mediated synthesis of COX-2 in brain cells. Lastly, the chemopreventive antioxidative agent selenomethionine attenuates COX-2 expression by interfering with NF-*κ*B-dependent transcription [35]. Altogether, there is considerable evidence that NF-*κ*B induction potentiates transcription of COX-2. It is important to note that particularly in bladder carcinogenesis, this regulatory coupling may underlie a cascade sequence: initially, cigarette smoke stimulates NF-*κ*B activity in bladder cells [18], leading to overexpression of COX-2, which in turn metabolizes inoffensive smoke substrates into active carcinogens. Among our other findings, we noted the presence of cytoplasmic staining of p65 in the majority of COX-2-negative cases [36]. This was not surprising, inasmuch as NF-*κ*B is ubiquitous and serves as a signal integrator, whose activation regulates the transcription of hundreds of genes, besides COX-2 [37].

## 4. Conclusions

Clearly the challenge for the future will be to determine molecular pathways to inhibit protumor functions of the inflammatory response while preserving the antitumor activities. Of course validation of these results in a larger number of cases, with detailed follow-up data and the use of supplementary in vitro studies are mandatory to verify our assumptions. The induction of COX-2 in combination with the antiapoptotic properties of NF*κ*B may contribute to the pathogenesis of UC. Celecoxib, a widely known selective COX-2 inhibitor [38], and curcumin, a plant extract with powerful NF-*κ*B-suppressing properties [39], are a promising treatment approach to the molecular pathology model described in the present study. The synergistic effect of the two agents, offering nontoxic celecoxib dosages, has been confirmed in colon cancer cells [40]. Still, further basic research to reveal every possible aspect of the network synthesized by key molecules, such as COX-2 and NF-*κ*B, may be the secret to a successful clinical outcome of cancer chemoprevention.

## Figures and Tables

**Table 1:** Clinical profile of the patients. (Figures in parentheses indicate percentages within histological types.)

Clinicopathologic parameters	*N*% (140p.)
Gender	
Male	94 (67%)
Female	46 (33%)

Age	23–90 (70 ± 10)

Normal cases	29 (20.7%)

Grade	
GI	27 (24.5%)
GII	30 (27%)
GIII	54 (48.5%)

T stage	
Ta	40 (36%)
T1	30 (27%)
T2	26 (23.4%)
T3	9 (8.1%)
T4	6 (5.4%)

**Table 2:** Correlation of tumor grade with COX-2 immunostaining. High grade of bladder carcinomas exhibited strong COX-2 cytoplasmic expression. (Spearman correlation coefficient *r* = 0.24, *P* *v* *a* *l* *u* *e* < .001).

Tumor grade	Negative	Weak	Moderate	Strong	Total (%)
Normal	6 (20.7%)	14 (48.3%)	4 (13.8%)	5 (17.2%)	29 (100.0%)
Grade I	3 (11.1%)	8 (29.6%)	9 (33.3%)	7 (25.9%)	27 (100.0%)
Grade II	1 (3.3%)	5 (16.7%)	16 (53.3%)	8 (26.7%)	30 (100.0%)
Grade III	5 (9.3%)	15 (27.8%)	23 (43.4%)	11 (18.9%)	54 (100.0%)

Total (%)	15 (10.7%)	42 (30.0%)	52 (37.1%)	31 (21.1%)	140 (100.0%)

**Table 3:** Expression of NF*κ*B, listed separately for cytoplasmic (C) and nuclear (N) localization, in tumor grade of bladder urothelial carcinomas (UC).

	Negative		Weak		Moderate		Strong		Total
	C	N	C	N	C	N	C	N	C-N
Normal	2(6.9%)	29(100%)	4(13.8%)	−(0%)	16(55.2%)	−(0%)	7(38.9%)	−(0%)	**29(100%)**
Grade I	0(0%)	0(0%)	3(11.1%)	7(26.0%)	8(29.6%)	5(18.5%)	16(59.3%)	15(55.5%)	**27(100%)**
Grade II	1(3.3%)	3 (10.0%)	7(23.4%)	5(16.7%)	9(30.0%)	13(43.3%)	13(43.3%)	9(30.0%)	**30(100%)**
Grade III	1(1.8%)	7(13.0%)	9(16.7%)	10(18.5%)	26(48.2%)	23(42.6%)	18(33.3%)	14(25.9%)	**54(100%)**

Total	4(2.9%)	39(27.9%)	23(16.4%)	22(15.7%)	59(42.1%)	41(29.3%)	54(38.6%)	38(27.1%)	**140**

**Table 4:** Coexpression of COX-2 and NF*κ*B of cytoplasmic (C) and nuclear (N). Positive correlation was established between expression of COX-2 and nuclear staining of NF*κ*B during progression of carcinogenesis.

Expression	Spearman correlation coefficient (*P* value)	Cochran-Armitage Test for trend *P* value
NF*κ*B (N) versus COX-2	0.11 (.208)	.160
NF*κ*B (C) versus COX-2	−0.01 (.900)	.920
